# Factors associated with low birth weight among neonates born at Olkalou District Hospital, Central Region, Kenya

**DOI:** 10.11604/pamj.2015.20.108.4831

**Published:** 2015-02-05

**Authors:** Onesmus Maina Muchemi, Elizabeth Echoka, Anselimo Makokha

**Affiliations:** 1 Field Epidemiology and Laboratory Training Program, Kenya; 2 Centre for Public Health Research, Kenya Medical Research Institute (KEMRI), Nairobi, Kenya; 3 Jomo Kenyatta University of Agriculture and Technology, Juja, Kenya

**Keywords:** Prevalence, factors, low birth weight

## Abstract

**Introduction:**

Ninety-two percent of Low Birth Weight(LBW) infants are born in developing countries, 70% in Asia and 22% in Africa. WHO and UNICEF estimate LBW in Kenya as11% and 6%by 2009 Kenya Demographic Health Survey. The same survey estimated LBW to be 5.5% in Central Province, Kenya. Data in Olkalou hospital indicated that prevalence of LBW was high. However, factors giving rise to the problem remained unknown.

**Methods:**

A cross-sectional analytic study was therefore conducted to estimate prevalence and distribution and determine the factors associated with LBW in the hospital. LBW was defined as birth of a live infant less than 2500g. We collected data using a semi-structured questionnaire and review of health records. A total 327 women were randomly selected from 500mothers. Data was managed using Epi Info 3.3.2.

**Results:**

The prevalence of LBW was 12.3% (n=40). The mean age of mothers was 25.6±6.2 years. Mean birth weight was 2928±533 grams. There were 51.1% (n=165) male neonates and 48.9% (n=158) females. The following factors were significantly associated with LBW:LBW delivery in a previous birth (OR=4.7, 95%C.I.=1.53-14.24), premature rapture of membranes (OR=2.95, 95%C.I.=1.14-7.62), premature births (OR=3.65, 95%C.I.=1.31-10.38), and female newborn (OR=2.32, 95%C.I.=1.15-4.70). On logistic regression only delivery of LBW baby in a previous birth (OR=5.07, 95%C.I.=1.59-16.21) and female infant (OR=3.37, 95%C.I.=1.14-10.00)were independently associated with LBW.

**Conclusion:**

Prevalence of LBW in the hospital was higher than national estimates. Female infant and LBW baby in a previous birth are independent factors. Local prevention efforts are necessary to mitigate the problem. Population-based study is necessary to provide accurate estimates in the area.

## Introduction

Low birth weight (LBW) is birth of a live infant less than 2500g (up to and including 2499g) irrespective of gestational age. It is often expressed as percentage of live born infants in a given time period. It can be subdivided into very low birth weight (less than 1500g) and extremely low birth weight (less than 1000g). Birth weight should be measured within the first hour of life before significant postnatal weight loss has occurred [1]. LBW is mainly as a result of preterm birth (before 37 weeks gestation) or due to restricted intrauterine growth [2]. According to WHO technical consultation report on promoting optimal fetal development, birth weight of an infant is dependent on amount of growth during pregnancy and the gestational age, and these factors are related to the genetic make-up of the infant and the mother, her lifestyle and her status of health [3].

The global prevalence of LBW is 15.5 percent, which amounts to about 20 million LBW infants born each year, 96.5 percent of them in developing countries. Half of all low birth weight babies are born in South-central Asia where 27 percent are below 2500g at birth while LBW levels in sub-Saharan Africa are estimated at 15 percent [1]. Most of the babies in Africa are at risk of being born preterm. The situation is different for South Asia where the rate of LBW is almost twice that of Africa but majority of the LBW babies are term babies who are small for gestational age. Preterm babies have a higher risk of death compared to full term babies [4].

The fourth Millennium Development Goal of the United Nations targets to reduce child mortality by two-thirds from 1990 to 2015. The under-five mortality and the infant mortality rates are the two indicators used to monitor this goal. The global under five mortality dropped by 47 percent from an estimated 90 to 48 deaths per 1000 live births between 1990 and 2012. Neonatal mortality declined from 33 per 1000 to 21 per 1000 live births over the same period. Although these indicators have shown a declining trend, the highest burden and the least improvement was observed in Sub-Saharan Africa compared to other regions of the world [5].

Neonatal mortality accounts for 40 percent of all deaths among children less than five years. Seventy-five percent occur during the first week of life, and between 25 to 45 percent occur within the first 24 hours. Preterm birth is the most common direct cause of newborn mortality. Preterm birth and being Small for Gestational Age (SGA) which are the reasons for low birth weight are important indirect causes of neonatal deaths. Low birth weight contributes 60 to 80 percent of all neonatal deaths [1]. Globally, the main causes of neonatal deaths are infections (35%), preterm birth (28%) and asphyxia (23%) [6]. There is substantial variation among regions on these three main causes. In Africa infections contributes 39%, prematurity 25% and asphyxia 24%. Low birth weight underlies majority of these deaths and links to maternal health, nutrition and infections such as Malaria and HIV. Similarly, in Kenya infections (25%), asphyxia (29%) and prematurity (34%) are the leading causes [4].

The prevalence of LBW in Kenya is estimated at 11 percent by WHO and UNICEF. The Kenya Demographic Health Survey of 2009 estimates LBW to be 6 percent [7]. LBW is a major cause of morbidity and mortality in Kenya. The current trends of infant and under five mortality rates in Kenya are declining. Under five mortality has declined by 36 percent from 115 per 1000 in 2003 KDHS to 74 deaths per 1000 in the 2008-09 KDHS, while infant mortality has declined by 32 percent from 77 deaths per 1000 in the 2003 KDHS to 52 deaths per 1000 in the 2008-09 KDHS. Neonatal mortality changed from 33 deaths per 1000 reported in the 2003 KDHS to 31 deaths per 1000 reported in the 2008-09 KDHS. The recorded decline of the infant and under five mortality is an indicator of progress in achieving the 4th millennium development goal [7]. However, neonatal mortality recorded a marginal decline compared to the other child health indicators. Addressing challenges associated with newborn deaths in Kenya has the greatest potential of contributing to this progress.

A number of factors have been identified to influence LBW. They include religious background, mother´s education, gestational age, mother´s weight, anemia, severe physical work, and tobacco chewing. In a study carried out at a tertiary care hospital in Uttar Pradesh, India, where 40% of mothers delivered low birth weight babies, Muslim mothers, mothers with no education, gestational age less than 37 weeks, mother´s weighing less than 50kg, hemoglobin less than 10gm/dl, severe physical work and tobacco chewing and history of abortion were found to be significant determinants of low birth weight [8].

Exposure to environmental pollutants including organophosphate pesticides have also been significantly associated with low birth weight deliveries. This was evident among Hispanic and African American pregnant women studied in New York City [9].

A study in Jimma zone South West Ethiopia found the following factors to be significantly associated with low birth weight. Mothers living in urban areas were found to be more likely to deliver low birth weights compared to their rural counterparts. The study related the association with urban residence to social lifestyles like heavy cigarette smoking and alcohol intake. Mothers who had experienced weight loss and those who had not had additional food during pregnancy had a significant increased risk of delivering low birth weight babies. Other factors including religion, ethnicity, history of a sexually transmitted infection, engaging in heavy work during pregnancy and history of chronic illness did not show any association [10].

The study by Siza 2008 found that mothers without formal education were 4 times more likely to give birth to low weight babies compared to those with formal education, whereas the father´s level of education significantly influenced the occurrence of low birth weight. In the same study unmarried mothers were found to be more likely to give birth to low birth weights compared to their married counterparts. Pregnancy and labor complications and illness during pregnancy were also significantly associated with low birth weight infants. These included; hypertension, pre-Eclampsia and Eclampsia disease complex, bleeding, placenta praevia, abruption placenta, premature rupture of membranes, anemia, Tuberculosis and Malaria in pregnancy. HIV positive women were twice more likely to give birth to low birth weight babies than HIV negative ones [11]. The HIV positive status also concurred with findings in a referral hospital in North West Ethiopia where HIV positive women were 3 times more likely to give birth to low birth weight infants than HIV negative ones [12].

In Kenya, factors documented to have significant influence on LBW can be summarized in two broad categories. The main factor associated with premature delivery is quality of antenatal care measured by timing, frequency of antenatal visits and tetanus injections. In addition, type of birth, birth order, region of residence and ethnicity influence premature delivery. Maternal nutritional status influences birth of small babies for gestational age. Other factors include maternal height and sex of the child. As a result shorter mothers tend to give birth to smaller babies, female babies are born smaller and multiple births are more likely to be smaller compared to single births. None of the socio-economic and cultural factors other than region were significant. Maternal age and birth order as risk factors of low birth weight have been identified in a number of population and hospital studies. In a cross-sectional analytic study that analyzed the 1993 Kenya Demographic Health Survey data, mothers aged below 20 years were found to have the smallest babies at birth. In the same study mothers aged 35 years and older had higher low birth weight babies compared to those aged 20-34 years. The distribution of low birth weights by birth order appeared to follow a similar pattern to maternal age. The highest proportion was identified among first order births with the smallest proportions reported among birth order two to five. However, after multilevel logistic regression analysis, only birth order showed significant influence on low birth weight. Quality antenatal and delivery care have been identified as important in preventing adverse pregnancy outcomes that include premature delivery, low birth weight, perinatal and maternal death. Although a strong association has been identified between premature deliveries and the baby´s size at birth, the two seem to be influenced by different sets of factors. Whereas the baby´s size at birth is influenced predominantly by maternal nutrition, premature delivery is predominantly influenced by the quality of antenatal care [13].

**Figure 1:**
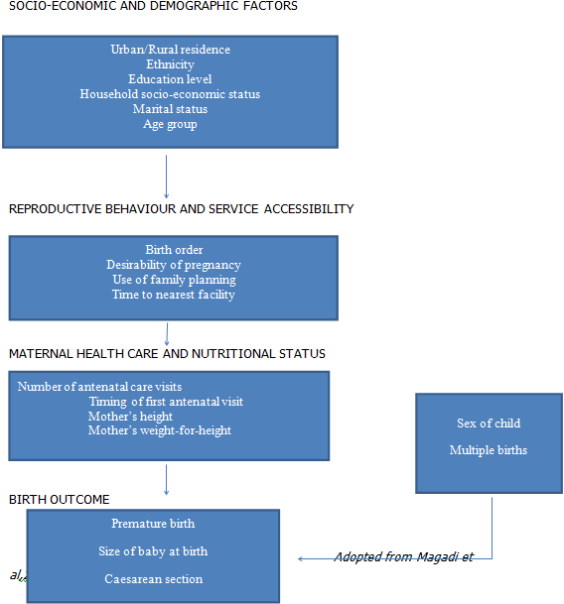
A conceptual framework visualizing the inter-relationships between potential risk factors and unfavorable birth outcomes

In a study that explored the pathways of the determinants of unfavorable birth outcomes, a number of factors were demonstrated to influence low birth weight indirectly through intermediate factors. Marital status, the desirability of pregnancy, use of family planning, and access to health services were demonstrated to be linked to low birth weight through antenatal care. The findings showed that antenatal care constituted the central link between many of the socio-demographic factors as well as reproductive factors with low birth weight. This may be an important explanation on the inconsistencies observed from previous studies regarding the relationship between these factors and low birth weight [14] Figure 1.

The contribution of low birth weight to neonatal morbidity and mortality in Kenya cannot be ignored. Neonatal intensive care is not readily available because of its initial and running costs and where available the bed capacity is extremely low. Even reasonably large rural district hospitals including those with pediatricians are poorly equipped to provide essential services to sick newborns and hence the need to implement simple, cost-effective and sustainable interventions to care for the special needs of newborns [15]. The need to focus on how to increase access to cost effective interventions that include control of the quality of infants born so as to decrease the burden and adopting simple strategies for the management of the high risk new born have been proposed [16]. Providing local solutions to public health problems have been found to be more acceptable and more likely to be implemented. The first steps entail identifying the problem.

The prevalence of low birth weights in Central region was documented as 5.5% in 2008-09 KDHS [7]. Although there was a marginal decline in neonatal mortality nationally, a comparative analysis of the KDHS 2003 and 2008-09 showed an increase of neonatal mortality by 15 percent from 27 to 31 per 1000 live births in central region [7]. The cause of this increase remains unknown, and the main causes of neonatal mortality are similarly unknown. Analysis of performance on Annual Operation Plan 6 (July 2010- June 2011) in the region revealed that Kiambu East and Nyandarua North districts had highest proportions of low birth weight infants at 13.1% and 8.6% respectively. Further analysis showed that Olkalou hospital contributed the highest burden in Nyandarua North district. Maternity records indicated that 126 out of 752 live births in the hospital between January 2013 and June 2013 constituted of LBW babies. This was approximately 16.8% prevalence. This study therefore sought to ascertain the prevalence of low birth weight, to describe the socio-demographic characteristics of women giving birth in the hospital and to investigate the factors contributing to the high prevalence of LBW in Olkalou district hospital. The findings from this study were intended to create awareness in the community about the problem and contribute towards formulating interventions to prevent low birth weight from a localized perspective. The findings will similarly be shared among various stakeholders to stimulate focused intervention programs to decrease the burden of the problem and to institute treatment strategies to care for babies with low birth weight.

## Methods

### Study design and area

We conducted an institution based cross-sectional study at Olkalou District Hospital, Central region, Kenya, from 28th October 2013 to 28th January 2014 among women delivering neonates during the study period. The study was undertaken in the postnatal ward. The hospital is a 142 bed public health facility situated in Nyandarua County, approximately 187 kilometers north of Nairobi (the capital city of Kenya). The hospital is a referral facility serving neighboring health institutions with catchment population of 70,273. The hospital delivery services are provided 24 hours. The hospital had one gynecologist, seven medical officers, fourteen nurses and intern clinical officers providing services in maternity.

### Sampling

The research study involved pregnant women presenting for labor and delivery at Olkalou hospital during the study period. The sample size was calculated using an assumed proportion (p) of 16.4% from the prevalence of LBW in a previous study conducted in Narok District Hospital in 2011 [17]. The Cochran formula (1963:75) was used to determine sample size [18]; n_0_ =Z^2^pq/e^2^, where n_o_= desired sample size; z= the standard normal deviate of 1.96, which corresponded to the 95% confidence level; p= (0.164), the proportion in the target population estimated to have LBW; 1-p= 0.836; e= degree of accuracy desired to get a 95% CI for a two-sided tails was 2.5%. The average number of deliveries per month was 167 (established in the facility). Three months were spent on the site, giving a total sampling frame of 500. Finite correction was done as appropriate using the following formula: n=n0/ (1+ (n0-1)/N), where n =sample size and N is the population size. Therefore n= 843/1+ (843-1)/500 = 314. A 10% contingency sample was added to cater for non-response giving a total sample size of 314+32= 346. The sample size for the study was therefore 346. A simple random method was used to draw a sample of 346respondents from the sampling frame of 500. Computer generated random numbers from Open Epi [19] were used. From day one of the study period, the clients were allocated positions consecutively as registration was done in the delivery register from position 1 to 500. The positions generated randomly by use of computer were arranged in ascending order by use of Microsoft Excel. Thus, every mother in the randomly selected position was recruited for the study. In situations where a respondent did not consent or did not meet the inclusion criteria, the respondent was replaced by the respondent in the consecutive position, after which the next random position was considered. This continued until the final sample size was obtained in the three months data collection period. The unit of study was all randomly selected live mothers who had given birth to a live neonate during the study period with the following exclusion criteria: multiple births, still births, maternal death following delivery, serious illness in which the mother was unable to respond, and situations where the mother was referred immediately following delivery.

### Study variables

The independent variables used in the study were selected on the basis of what was found in published literature regarding their influence on low birth weight. They included age, residence, mother´s education level, partner´s education level, employment status of mother and spouse, place of employment, religious background, marital status; age at first birth, number of previous births, number of pregnancies, preceding birth interval, family size, desired family size, desirability of the pregnancy, family planning practice, last baby weight, bad obstetric history (BOH), previous history of low birth weight or prematurity, previous history of neonatal death, previous surgery on uterus and cervix; time to maternity health facility; timing of first ANC visit, number of ANC visits, tetanus immunization; history of tobacco/marijuana, history of alcohol consumption, history of exposure to agricultural spraying, source of water; history of sexually transmitted infection, malaria, HIV/AIDS, Syphilis, and Tuberculosis, chronic conditions e.g. Diabetes Mellitus, hypertension, heart disease, respiratory disease and renal disease; hemoglobin level, maternal weight and height, body-mass index, mid-upper arm circumference, additional food during pregnancy, Iron and Folate supplementation, avoidance of food in pregnancy; gestational age at delivery, sex of newborn and congenital malformation. In order to determine the association between these factors and LBW in Olkalou hospital, a conceptual framework similar to that described by Magadi was adopted [14] Figure 1.

### Data collection

An elaborate semi-structured interviewer administered questionnaire was used to collect data. Prior, we pretested among ten conveniently selected subjects in Karatina District Hospital postnatal ward. We designed the questionnaire in English, but we administered to the respondent in *Kikuyu* (local language), *Kiswahili*, or English subject to the choice of the client. Once the client was selected, she was interviewed in a private room in the maternity unit so as to ensure privacy. Mothers who had undergone caesarian section were not examined until after 48-72 hours. The room was positioned further from the busy side to avoid interruption. A note was fixed on the outside to indicate that an interview was in progress. Review of records and anthropometric measurements were similarly done in the same room. Gestational age was determined using the mother´s recorded or reported Last Normal Menstrual Period and the date of delivery. Where unknown, any ultrasound report was used to confirm gestation, otherwise gestation was regarded as unknown. Previous history of LBW and premature births was assessed by asking the mother if she had delivered ‘small’ or ‘very small baby’ or had given birth to a baby before term. A confirmation was made by checking the mother and child health booklet. We assessed the dietary history by asking the mother to describe the food she had eaten the previous day before admission to hospital. Other questions included whether she had had additional food during pregnancy, any nutritional problems or if any health worker had counseled her on the importance of good nutrition during pregnancy.

We reviewed labor and delivery notes, mother and child health booklet, and the delivery register to ascertain obstetric and gynecologic history, antenatal care, health issues during pregnancy, labor, delivery, clinical care and the outcome of birth. The records review took place during the postnatal period after other essential procedures to the client had been completed or just before discharge to minimize interruption. A study subject number was used as the unique code for the client.

We measured neonatal weight using a standard beam balance (Crown), within one hour upon delivery. The mother was weighed using a standard weighing machine (Seca). We requested the respondent to remove extra clothes, remove their shoes and step on a zeroed weighing scale. Weight was recorded to the nearest 0.1kg. The height was measured using a Height board. We asked the participant to stand without shoes in front of the height board, with the head erect and the arms hanging naturally at the sides. Height was recorded to the nearest 0.1cm. The mid-upper arm circumference (MUAC) was measured using a flexible non-stretchable standard tape measure. The circumference was located and measured at the mid-point between the tip of the acromion process of the scapula and olecranon process of the ulna. For right-handed women the circumference of the left upper arm was measured while for left-handed women, the right arm was used instead. We measured the arm while hanging down at the side and relaxed. The MUAC was recorded to the nearest 0.1cm. We considered a MUAC of 23cm as the cut-off point.

Two midwives experienced in delivery care were recruited to assist in accurate weighing of the newborns and measuring the weights and heights of mothers and the mid-upper arm circumference. Consequently, they were trained by the investigator for eight hours on the purpose and objectives of the study, the standard procedures of weight measurement, height measurement, the mid upper-arm circumference and the interviewing technique. They were further trained on the importance of maintaining confidentiality and obtaining consent before interviewing or obtaining anthropometric measurements, being patient, understanding, respectful and genuine when handling the respondents. The investigator interviewed the mothers and supervised the midwives while taking the anthropometric measurements.

### Data analysis

Data was entered and analyzed using Epi Info statistical software version 3.3.2. Descriptive analysis was done to determine the prevalence of low birth weight and to determine the socio-demographic characteristics of women delivering in Olkalou District Hospital. Bivariate, stratified and logistic regression analysis were performed to determine the association between the independent and the outcome variable and to control for potential confounders. A two-tailed test of significance was used. The measures of association were reported with a 95% confidence interval. Factors with a p-value of

### Human subjects

We obtained ethical clearance for the research from the Kenyatta National Hospital research and ethics committee. We also obtained permission from Nyandarua County Health Services Director and the Medical Superintendent of the hospital to undertake the study.

The purpose of the study was explained to the mothers and subsequently consent was obtained for participation in the study. The respondent mothers were interviewed and examined in a private room. No name was recorded on the questionnaire or any other identifier relating to the respondent. A study subject number representing the randomly selected position of the subject was used as the unique identifier.

## Results

### Socio-demographic and reproductive characteristics of respondents

A total of 327 neonate/mother pairs participated in the study constituting a response rate of 94.5%. A total of 19 clients were excluded due to having delivered a still birth, or having had a multiple delivery. Of these 327 sampled respondents, 40(12.3%) gave birth to low birth weight neonates. The mean age of the respondents was 25.6±6.2 years. Thirty seven (11.3%) of the sampled women were urban or peri-urban. One hundred and forty one (48.1%), the majority had completed secondary education while 134 (45.7%) were of primary education, 14(4.8%) were of tertiary education, while 4(1.5%) had had no formal education. Similarly, among their partners, 53.9% (n=132) had completed secondary education, 35.5% (n=87)were of primary education, while 10.6% (n=26) were in tertiary level of education. Majority (46.4%, n=135) were self-employed followed by those unemployed (39.2%, n=114). In contrast, majority (64.1%, n=157) of their male partners were self-employed, 77(31.4%) were employed, 10(4.1%) were unemployed, while only 1(0.4%) was a student. Two hundred and twenty nine (79.5%) were protestant, 55 (19.1%) were Catholic, while 4 (1.4%) could not identify with a religious background. Two hundred and sixty forty (81.2%) of the mothers were married, 51(15.7%) were single, 5 (1.5%) had either separated or divorced, while a similar proportion were widowed Table 1.

**Table 1:** Socio-demographic characteristics of women delivering at Olkalou hospital, 28th October to 28th January 2014

Demographic characteristics	Variable	n (%)
Age in years	<20yrs	51 (15.6)
	20-35yrs	243 (90.2)
	>35yrs	32 (9.8)
Residence/village/estate	Urban (Olkalou)	37 (11.3)
	Rural	290 (88.7)
Level of education of mother	No formal education	4 (1.4)
	Primary education	134 (45.7)
	Secondary education	141 (48.1)
	Tertiary education	14 (4.8)
Partner´s education level	No formal education	0 (0)
	Primary education	87 (35.5)
	Secondary education	132 (53.9)
	Tertiary education	26 (10.6)
Mother´s employment status	Employed	31 (10.7)
	Self-employed	135 (46.4)
	Student	11 (3.8)
	Unemployed	114 (39.2)
Partner´s employment status	Employed	77 (31.4)
	Self-employed	157 (64.1)
	Student	1 (0.4)
	Unemployed	10 (4.1)
Religion	Protestant	229 (79.5)
	Catholic	55 (19.1)
	Unknown	4 (1.4)
Marital status	Single	51 (15.7)
	Separated/divorced	5 (1.5)
	Married/cohabiting	264 (81.2)
	Widowed	5 (1.5)

The sampled mothers had the following reproductive characteristics. The mean age at first birth was 20.2±3.2. The mean gravidity was 2.5±1.6. Majority (59.2%, n=189) were less or equal to gravida 2. The mean birth interval was 2.8±2.4years. Those with a birth interval greater than 2 years were more (53.2%, n=125). The mean parity was 2.3±1.5, with majority (62.5%, n=196) having had one or two living children. The mean desired family size was 3.4±1.2. Majority (58.4%, n=170) desired a family size of three or more children. Most (81.8%, n=239) of the mothers had intended to have their current pregnancy. However it is important to note that 53(18.2%) had either desired to have the pregnancy later or did not intend to have the pregnancy at all. The mean weight of the previous baby was 2932±531, with 18(10.5%) reporting having had a low birth weight baby in their previous pregnancy. Twenty four (8%) clients reported having had a previous abortion, 20(6.8%) reported a history of low birth weight or prematurity, 9(3.1%) reported history of neonatal death, while 22(7.5%) reported having had a previous surgery of the uterus or the cervix. Majority of the mothers (42.9%, n=133) had attended 3 antenatal visits, the average being 3±1.1. Most mothers (71.3%, n=21) had attended more than 2 antenatal visits. Two hundred and fifty five (87%) had received anti-tetanus immunisation during the pregnancy.

Thirty one (10.5%) of the sampled mothers had been referred from another facility, majority (79.3%, n=23) having been referred from a health centre, 4(13.8) from a dispensary and 2(6.9%) from a faith based facility. The onset of labour was mainly spontaneous, constituting 94.0% (n=283). Twelve (4%) had been induced while 6(2%) had not established labour at the time of delivery. The main mode of delivery was spontaneous vertex delivery (88.3%, n=288), followed by caesarean section (10.7%, n=35) while 0.9% (n=3) were delivered through assisted breech. The deliveries were mainly conducted by a nurse midwife (82.8%, n=265), followed by the medical officers (11.3%, n=36). Sixteen (5.0%) were conducted by clinical officer interns, while 2(0.6%) were conducted by a medical officer intern, with 1(0.3%) being conducted by an obstetrician. Majority of the mothers (82%, n=219) were 37-42weeks gestation at the time of delivery, 27(10.1%) were more than 42 weeks, while 21(7.9%) were less than 37 weeks. For those who had caesarean section performed, the main reason was obstructed labour and cephalopelvic disproportion (CPD) at 27.3% (n=9) followed by previous scar (24.2%, n=8) and foetal distress (21.2%, n=7) Others included delayed second stage (12.1%, n=4), ante-partum haemorrhage (6.1%, n=2), bad obstetric history, cord prolapse and premature rapture of membranes (PROM) were each 3.0% (n=1). The mean Apgar score was 8±1.2. Eleven (3.5%) neonates had an Apgar score of less than 6. The mean birth weight was 2928±533 grams. There were 51.1% (n=165) male neonates and 48.9% (n=158) were females. Ten (3.1%) of the new-borns had a birth defect Table 2.

**Table 2:** Reproductive and obstetric characteristics of women delivering at Olkalou district hospital, 28th October to 28th January 2014

Factors	Variable	n (%)
Age at first birth	<15yrs	5 (1.7)
	15-19yrs	124 (41.6)
	≥20yrs	169 (56.7)
Number of previous births	<2	180 (57.0)
	2-4	118 (37.3)
	>4	18 (5.7)
Pregnancy interval of previous birth	<2yrs	12 (6.6)
	2-4	123 (67.6)
	>4	47 (25.8)
Family size (living children)	2 or less	192 (62.6)
	3 or more	117 (37.4)
Desired family size	2 or less	49 (16.8)
	3 or more	242 (83.2)
Desirability of current pregnancy	Yes	239 (81.8)
	Later	49 (16.8)
	No more	4 (1.4)
Family planning practice	Used modern methods	158 (54.3)
	Never used any method	128 (44.0)
	Traditional methods	5 (1.7)
Last baby birth weight	<2500g	18 (10.4)
	>2500g	155 (89.6)
Timing of 1^st^ ANC visit	1^st^ trimester (≤12weeks)	1 (0.4)
	2^nd^ trimester (12-24)	184 (64.8)
	3^rd^ trimester(≥24weeks)	99 (34.9)
Number of ANC visits	2 or less	82 (27.1)
	3 or more	221 (72.9)
Gestation	<37weeks	21 (7.9)
	37-42	219 (82.0)
	>42	27 (10.1)
Onset of labor	Spontaneous	283 (94.0)
	Induced	12 (4.0)
	No labor	6 (2.0)
Assistance during delivery	Midwife	265 (82.8)
	Medical officer	36 (11.3)
	Clinical officer	16 (5.0)
	MO intern	2 (0.6)
	OBGYN specialist	1 (0.3)
Mode of delivery	Spontaneous	288 (88.3)
	Caesarian section	35 (10.7)
	Assisted breech	3 (0.9)
Birth weight	<2500g	40 (12.3)
	≥2500g	285 (87.7)

MO-medical officer; OBGYN- Obstetrician gynecologist

The mothers had the following health and nutritional characteristics; on average it took approximately 1 hour for them to reach a maternity health facility (mean 63.2±51.9 minutes). In general, slightly more than half the mothers (52.6%, n=154) obtained domestic water from a well or borehole followed by tap water (29.4%, n=86). Approximately a fifth (19.8%, n=58) reported having been exposed to agricultural spraying during pregnancy. None reported having smoked or taken illegal drugs. Only 1(0.3%) had consumed alcohol. Maternal obstetric and medical illnesses were assessed. Among women with medical and obstetric problems, 1(0.3%) had had a sexually transmitted infection, 3(1.0%) had been treated of malaria, 9(3%) were HIV positive, 2(0.7%) had been tested VDRL positive and none had been diagnosed of Tuberculosis. Thirteen (4.4%) had a chronic disease, 6(2%) had pre-Eclampsia or Eclampsia, 5(1.7%) had experienced vaginal bleeding during pregnancy and 26(8.9%) had raptured membranes prematurely.

The following were the means of the nutritional parameters that were measured: haemoglobin level 12.6±1.2g/dl, height 178±0.8cm, and weight 62.8±8kg. Thirty nine (11.9%) of the women were undernourished ((MUAC<23cm), while 77.4% (n=253)) received iron and folic acid supplements. The main nutritional complaints were nausea and vomiting (36.7%, n=120), heart burn (27.8%, n=91) and poor appetite (19.3%, n=63). Sixty three (21.7%) reported having avoided certain foods during pregnancy. The main reason for this avoidance was nausea and vomiting (58.1%, n=36), heart burn (16.1%, n=10) and poor appetite (11.3%, n=7). Only 4.8% (n=3) reported avoidance of eggs due to myth of giving birth to a big baby Table 3.

**Table 3:** Health and nutritional characteristics of women delivering at Olkalou district hospital, 28th October to 28th January 2014

Factors	Variable	n (%)
Time to maternity facility	< 1 hour	147 (37.7)
	≥ 1 hour	150 (52.3)
Source of water	River	27 (9.2)
	Tap water	86 (29.4)
	Well/borehole	154 (52.6)
	Harvested water	26 (8.9)
Hemoglobin level	Normal(≥10)	259 (97.0)
	Mild (8.1-9.9g/dl)	8 (3.0)
BMI	<18.5	46 (15.9)
	≥18.5	244 (84.1)
Mid-upper arm circumference	<23cm	39 (14.0)
	≥23cm	240 (86.0)
Food source	Garden	203 (62.1)
	Market	98 (30.0)
	Donation	1 (0.3)
Nutritional problems	Nausea and vomiting	120 (36.7)
	Heartburn	91 (27.8)
	Poor appetite	63 (19.3)
	Constipation	30 (9.2)
	Pica	11 (3.4)
	Muscle cramps	3 (0.9)
	None	47 (14.4)

### Factors associated with low birth weight

The results of bivariate analysis indicated a significant association between the weight of the previous baby, premature rapture of membranes, premature birth, Apgar score of the newborn, female infant and low birth weight. Mothers who had delivered a low birth weight baby in their previous pregnancy were almost 5 times more likely (OR= 4.7, 95%C.I.= 1.53-14.24, p-value=0.01) to give birth to a LBW baby compared to those who had given birth to a normal weight baby. Additionally, premature rapture of membranes was also one of the risk factors (OR=2.95, 95%C.I.=1.14-7.62, p-value=0.04). There was a statistically significant difference in the birth weights between mothers who gave birth in less than 37 weeks gestation (OR=3.68, 95%C.I.=1.31-10.38, p-value=0.02) and those that delivered at 37 or more weeks gestation. Newborns with an Apgar score of less than six (asphyxia) had sevenfold (OR=7.03, 95%C.I.=2.03-24.35,, p-value<0.01) likelihood of having been born LBW compared to those that were born with a score of 6 or more on Apgar. Female newborns had lower birth weight than males (16.6% vs 7.9%). The association between female neonate and LBW was statistically significant in the bivariate analysis (OR= 2.32, 95%C.I.=1.15-4.70, p-value=0.03). Below is summary table of the factors that were identified significantly associated with low birth weight p-value=0.03 Table 4.

**Table 4:** Factors associated with low birth weight among neonates born at Olkalou District Hospital, 28th October 2013 to 28th January 2014

Risk factor	Variable	OR	95% C.I.	P-value
Previous baby birth weight	<2500g	4.7	1.53-14.24	0.01
	≥2500g			
Premature rapture of membranes	Yes	2.95	1.14- 7.62	0.04
	No			
Gestation age at delivery	<37weeks	3.68	1.31-10.38	0.02
	≥37 weeks			
Apgar score	<6	7.03	2.03-24.35	<0.01
	≥6			
Infant sex	Female	2.32	1.15-4.70	0.03
	Male			

P-value if <0.05, then the association between the factor and the Low Birth Weight was statistically significant. OR- odds ratio

### Confounding and effect modification

Results from stratified analysis showed that infant sex and residence were confounders while maternal age was found to be an effect modifier.

### Independent factors associated with low birth weight

Twelve factors were taken to the unconditional logistic regression model using a backward building strategy. All variables which had a p-value less than 0.1 were included in the model. These included gestation at delivery, birth weight of previous baby, premature rapture of membranes, Apgar score. Infant sex and residence were added as confounders while maternal age was included as an effect modifier. The final model showed that only two factors were independently associated with low birth weight at Olkalou District Hospital; low birth weight of previous baby (OR=5.07, 95%C.I.=1.59-16.21, p-value Table 5.

**Table 5:** Independent factors associated with low birth weight among neonates born at Olkalou District Hospital, 28th October to 28th January 2014

Term	Odds Ratio	95%	C.I.	Coefficient	S. E.	Z-Statistic	P-Value
Female infant (Yes/No)	3.3724	1.1378	9.9954	1.2156	0.5544	2.1928	0.0283
LBW of Previous baby (Yes/No)	5.0733	1.5882	16.2063	1.6240	0.5926	2.7406	0.0061
CONSTANT	*	*	*	-3.0569	0.4959	-6.1649	0.0000

## Discussion

The prevalence of low birth weight in the hospital (12.3%) was higher than the national 2009 KDHS value of 6.0% and 5.5% value for Central region [7]. However, this was not a population based study. A study in Nyanza Provincial General Hospital, Kenya recorded a prevalence of 15.0% [16]. The prevalence was lower than the 16.4% prevalence documented in Narok District Hospital of Rift Valley province of Kenya [17]. This prevalence was similarly lower than the prevalence of 22.5% in Jimma zone, South West Ethiopia [10], 13.6% recorded in Kilimanjaro Christian Medical Centre (KCMC) referral Hospital in Moshi, northern Tanzania [11] and 17.1% documented in Gondar University Hospital of North West Ethiopia [12]. However, the prevalence was higher than the 9.1% documented in facility based retrospective study conducted in MCH clinics in three facilities in Korogwe district, Tanzania [20]. The prevalence in Olkalou hospital could be attributed to the fact that it is a referral hospital in Nyandarua County.

The findings in this study show that most of the women who gave birth at Olkalou district hospital were of the 20-35 years age group. This is the recommended reproductive age group. This was consistent with findings documented elsewhere [10–12, 14, 20]. Majority of the mothers were of rural residence. This was expected as it was predominantly a rural setting. Same findings were documented in Jimma Zone, South West Ethiopia [10]. Most sampled women had completed secondary education. This was similar with the findings in Gondar University hospital, Ethiopia where respondents with secondary and tertiary education were equal in proportion [12]. The difference could have been due to the fact that the sampled population in Ethiopia was predominantly urban and therefore likely to have had a higher education status. However, findings from other studies found majority of the respondent mothers to have been of primary education [10, 14]. In this study, the women were mainly self-employed similar to their partners. This was expected due to the fact that the main economic activity in the area is farming. The respondents were mostly married similar to findings in other settings [10, 12, 14] and of protestant faith.

In the bivariate analysis, among the reproductive and obstetric factors, a significant association was found between low birth weight and having delivered a LBW baby in the previous birth, premature rapture of membranes, premature births, Apgar score of less than 6 and female infant. This was similar to other studies [8, 10, 12]. After unconditional logistic regression low birth weight of previous birth and female infant were independently and significantly associated with low birth weight. The other potential factors included in the study such as socio-demographic factors, access to health services, maternal health status including nutritional status, maternal health lifestyle and exposure to pollutants failed to be associated with low birth weight.

The study experienced a number of limitations. Being a cross-sectional study, it was not possible to show seasonal variation in low birth weight. The study is hospital based, and therefore it was not possible to generalize the results to a particular population as compared to population based studies. The hemoglobin level used to assess anemia as a risk factor in this study was what was available in record. Owing to the fact that mothers attend ANC clinic at different gestation in their pregnancy, it would have been more scientifically logical to follow the pattern of hemoglobin level from antenatal period to delivery in estimating the association of anemia and low birth weight. The Last Normal Menstrual Period (LNMP) was obtained from the mother child booklet, the delivery register or asking the mother. The LNMP was then used to calculate gestational age. For those mothers whose LNMP was recorded unknown or could not recall and no ultrasound record was available, gestation age could not be determined and was therefore regarded as non-response. However, an association was calculated to determine if there was a relationship between those with unknown gestation age and low birth weight.

Despite the limitations, this study has made an important contribution on the prevalence of low birth weight in Olkalou district hospital, the socio-demographic and the reproductive characteristics of women delivering in the hospital. In particular, the study has identified the important risk factors that may contribute to the occurrence of high prevalence of low birth weight in the hospital.

## Conclusion

The prevalence of low birth weight in Olkalou District Hospital was 12.3%. There are no documented cut-off values of public health significance for low birth weight in Kenya or internationally. However, 12.3% prevalence represents a substantial risk to neonatal death among newborns in this hospital. It is important therefore that the newborn unit is well equipped to provide essential services to newborns at risk, including low birth weight. In this study, low birth weight births were more likely to occur among women who had delivered a low birth weight baby in the previous birth, among those who had raptured membranes prematurely, those who had given birth prematurely, and among those who gave birth to female infants. Low birth weight baby in the previous birth and female infant were identified as independent factors associated with low birth weight in Olkalou District Hospital. Whereas being female infant is a non-modifiable factor because it is biological and inherent, giving birth to a low birth weight baby in a previous birth can be altered to reduce the occurrence of low birth weight in the hospital. Asphyxia was more likely to occur among the low birth weight infants. Although the indicators of quality antenatal care, such as timing of first antenatal visit, number of antenatal visits and tetanus injections were not significantly associated with low birth weight, it is important to note that the association between low birth weight of previous birth, premature rapture of membranes and premature delivery are predominantly linked to the quality of antenatal care. **Recommendations:** retraining and mentorship on focused antenatal care is necessary to ensure risk of low birth weight is detected early and treated appropriately. We recommend a population based study to ascertain the prevalence of low birth weight and associated risk factors in Nyandarua County.
